# The Role of Morphology and Phase Engineering in Molybdenum Sulfide for Robust Photoelectrochemical Hydrogen Evolution on III–V Photoelectrodes

**DOI:** 10.1002/cssc.71110

**Published:** 2026-09-26

**Authors:** Zakaria Anfar, Fatima Merhi, Bruno Fabre, Gabriel Loget, Yoan Léger, Nicolas Bertru, Charles Cornet, Chrystelle Salameh, Damien Voiry

**Affiliations:** ^1^ Institut Européen des Membranes (IEM) UMR 5635 CNRS ENSCM Université de Montpellier Montpellier France; ^2^ Institut des Sciences Moléculaires (ISM) UMR 5255 CNRS Bordeaux INP Université de Bordeaux Pessac France; ^3^ Institut des Sciences Chimiques de Rennes (ISCR) UMR 6226 CNRS Université de Rennes Rennes France; ^4^ Institut FOTON UMR 6082 CNRS INSA Rennes Université de Rennes Rennes France

**Keywords:** functionalization, GaAs, hydrogen, MoS_2_, photoelectrocatalysis

## Abstract

Solar‐driven hydrogen production via photoelectrochemical (PEC) water splitting offers a direct route to decarbonized fuels without grid dependence. Although III–V semiconductors exhibit exceptional optoelectronic properties, their reliance on platinum group cocatalysts limits scalability. Here, we report a platinum group metal‐free (PGM‐free) hybrid photocathode integrating an epitaxially grown GaAs/Si:p junction with mixed‐phase MoS_2_ nanostructures for efficient hydrogen evolution in acidic media. Through systematic control of morphology, crystallinity, and phase composition, we demonstrate that 1T′‐rich MoS_2_ nanoflowers outperform exfoliated nanosheets, bulk 2H‐MoS_2_, and amorphous powder MoS_*x*_. The nanoflower architecture delivers higher saturation photocurrent densities and a positive shift in onset potential, attributed to enhanced charge transport, catalytic kinetics, and light penetration, while simultaneously improving stability. Notably, the hierarchical nanoflower morphology and 1T′ phase synergistically enhance photocurrent performance. The optimized GaAs/Si:p photocathodes modified with MoS_2_ nanoflowers exhibit stable operation with a Faradaic efficiency of ~97% for hydrogen evolution. These findings establish morphology‐phase synergy as a critical design principle for developing PGM‐free PEC systems, paving the way for cost‐effective solar fuel production.

## Graphical abstract

Morphology and phase engineering of MoS_2_ cocatalysts strongly influence the performance of GaAs/Si:p photocathodes. 1T′‐rich MoS_2_ nanoflowers combine favorable charge transport, light penetration, and catalytic activity, enabling robust photoelectrochemical hydrogen evolution.

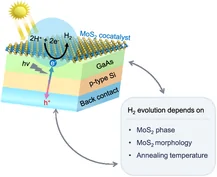

## 1 Introduction

Converting solar energy into chemical bonds offers a clean, scalable, and sustainable energy solution [1, 2]. Among the most promising avenues in this field is the production of green hydrogen (H_2_), a carbon‐neutral fuel with exceptional energy density [3, 4]. Photoelectrochemical (PEC) water splitting offers a direct, solar‐driven route to hydrogen production (e.g., in acidic media: 2H^+^ + 2e^−^ → H_2_) [5, 6]. This process not only circumvents the need for external electrical input but also aligns with global efforts to decarbonize energy systems. Recent advances have demonstrated that epitaxially grown GaAs thin films (TFs) on p‐doped silicon (Si:p) substrates can achieve remarkable H_2_ yields in alkaline media when paired with Ni catalysts [7]. The efficiency of such systems is critically dependent on the integration of high‐performance cocatalysts that minimize the required photovoltage for hydrogen evolution [6]. Therefore, the development of heterojunctions with well‐defined interfacial contact can promote efficient charge separation and improve hydrogen evolution performance [8]. However, despite major efficiency gains, PEC durability remains limited by semiconductor corrosion during aqueous water splitting [9, 10, 11, 12, 13, 14].

We previously developed Ni‐modified GaAs/Si:p photocathodes for PEC in alkaline media. The devices remained stable for several hours and achieved ∼100% Faradaic efficiency for H_2_ [7]. Alternatively, platinum and other noble metals show exceptional hydrogen production activity owing to their near‐zero overpotential and outstanding catalytic activity [15, 16]. However, their high cost and limited availability pose significant barriers to large‐scale deployment. This has prompted intensive research into earth‐abundant alternatives, particularly two‐dimensional materials, which combine high surface areas with tunable electronic properties [17]. Metal sulfide nanomaterials have emerged as particularly attractive cocatalysts, as they can simultaneously protect Si‐ and III–V‐based photoelectrodes against corrosion while efficiently catalyzing the hydrogen evolution reaction (HER), especially in acidic media [16]. In particular, transition metal chalcogenides, such as MoS_*x*_ (with *x* ≥ 2), have been extensively investigated as HER catalysts under acidic conditions [18, 19]. Over the past decades, extensive investigations have elucidated the influence of phase, defect density, exfoliation degree, and interfacial modulation on the catalytic performance of MoS_2_ [20, 21, 22]. Yet, linking intrinsic HER activity to PEC performance remains challenging because catalyst morphology, optical absorption, charge transfer kinetics, and semiconductor/catalyst interfacial properties are closely intertwined.

In this present work, we systematically investigate the photocatalytic properties of GaAs/Si:p photocathodes modified with molybdenum sulfide cocatalysts as PEC water splitting systems. By methodically tuning the crystalline structure, morphology, and annealing conditions of MoS_*x*_ systems, we engineered a series of hybrid photocathodes and rigorously characterized their performance. We demonstrate that 1T′/2H‐MoS_2_ nanoflowers possessing a high surface area significantly enhance PEC activity in acidic media. To further elucidate the role of crystallinity, we examined its effects for electrodeposited MoS_*x*_ subjected to thermal annealing. This work advances our fundamental understanding of the synergistic interplay between catalyst morphology, phase composition, and structural properties while offering practical design principles for the development of efficient, earth‐abundant photocathodes for sustainable solar hydrogen production.

## 2 Results and Discussion

### 2.1 Performance of MoS_2_ Nanoflower Cocatalysts

**FIGURE 1:**
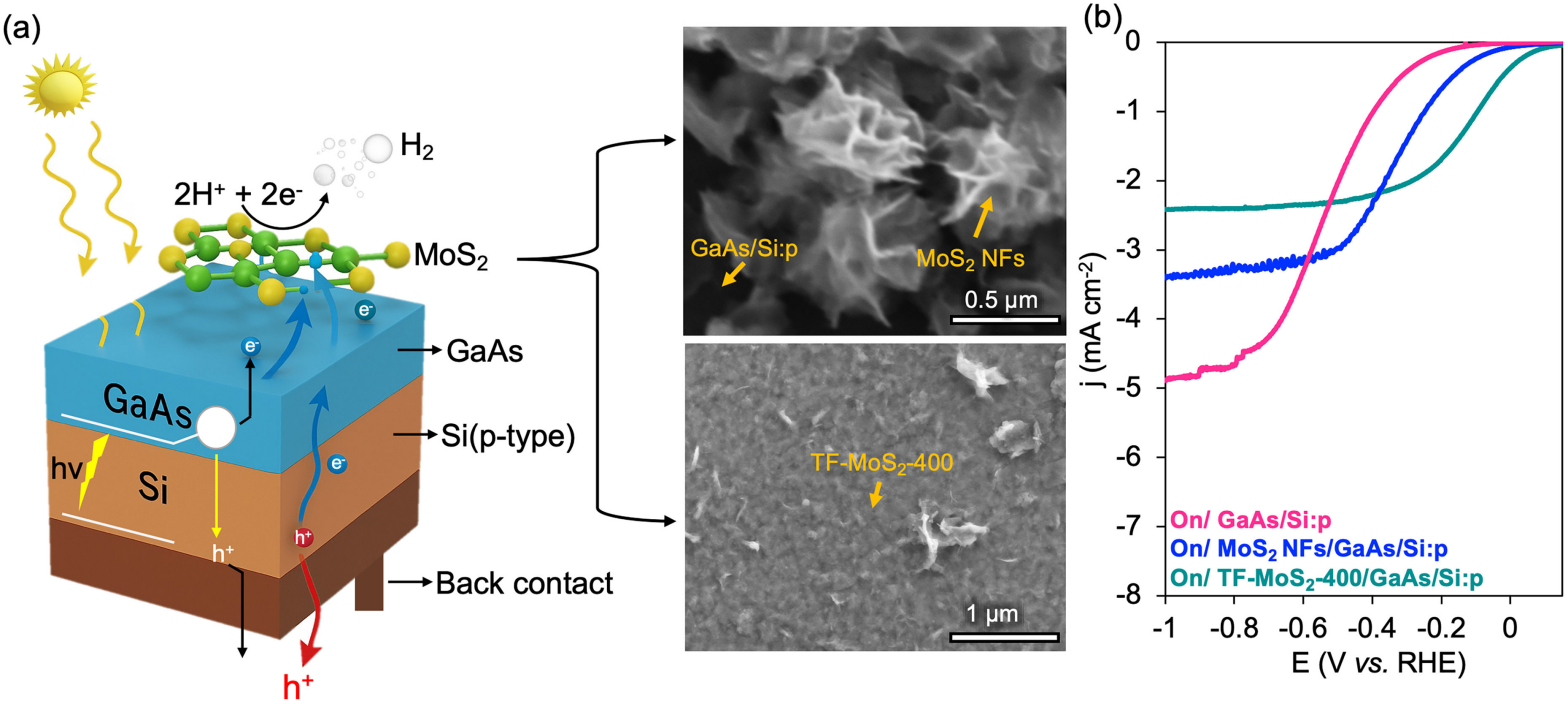
Photocathode design and PEC performance. (a) Schematic illustration of the photocathode architecture and charge separation mechanism in 0.2 M H_2_SO_4_ using hybrid MoS_2_/GaAs/Si:p cocatalysts. (b) LSV curves of GaAs/Si:p, MoS_2_ NFs/GaAs/Si:p, and TF‐MoS_2_‐400/GaAs/Si:p under simulated solar illumination.

Hybrid photocathodes were prepared by depositing molybdenum sulfide catalysts into GaAs grown by molecular beam epitaxy on a Si:p substrate (GaAs/Si:p) [7, 23, 24]. The GaAs/Si:p junction facilitates light absorption and effective charge separation, while molybdenum sulfide enables efficient electron transfer, photocorrosion resistance, and acts as a hole blocker, serving as a cocatalyst for the HER (Figure 1a). We synthesized crystalline MoS_2_ nanoflowers (MoS_2_ NFs) via a hydrothermal route [25, 26] (Figure S1a), which were subsequently spray‐coated into the GaAs/Si:p surface (Figure S1b). The MoS_2_ NFs were intercalated during the synthesis with (R)‐(+)‐α‐methylbenzylamine (MBA) to increase the interlayer distance, stabilize the metastable 1T′ phase [26], and therefore the intrinsic specific surface area. We first assessed the impact of MoS_2_ NFs cocatalysts on the photoelectrode’s performance in 0.2 M H_2_SO_4_ electrolyte under an AM 1.5G‐filtered simulated sunlight. For comparison, TFs of MoS_2_ were prepared by electrodeposition of MoS_*x*_, followed by annealing at 400 °C under argon/H_2_ (Figure S1c). Before performing the linear sweep voltammetry (LSV), repeated cycling using cyclic voltammetry is done to electrochemically stabilize the photoelectrodes (Figure S2a). The bare GaAs/Si:p photocathode exhibited a saturation current density (*j*_sat_) of approximately −5 mA cm^−2^ (Figures 1b, S2b). The use of MoS_2_ NFs resulted in a positive shift in the onset potential (*V*_onset_), albeit with a slight reduction in *j*_sat_ compared to the bare photocathode. In comparison, MoS_2_ TFs (TF‐MoS_2_‐400) demonstrated improved *V*_onset_ to approximately +0.1 V versus reversible hydrogen electrode (RHE) but a significantly reduced value of *j*_sat_ (Figure 1b). The superior *V*_onset_ of MoS_2_ TFs suggests improved interfacial and electronic coupling between the electrodeposited MoS_2_ and the GaAs/Si:p substrate compared to MoS_2_ NFs. This observation is further supported by electrochemical impedance spectroscopy (EIS) measured at 0 V versus RHE (Figure S3).

**FIGURE 2:**
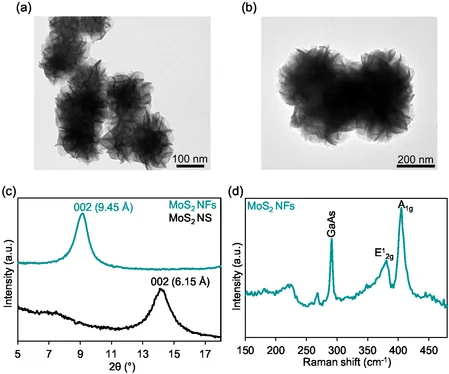
Structural and chemical characterization of MoS_2_ nanoflowers. (a) Large area and (b) magnified TEM images showing the flower‐like morphology of MoS_2_ NFs. (c) X‐ray diffraction patterns showing the interlayer expansion of MoS_2_ NFs compared to conventional MoS_2_ nanosheets (NS) and (d) Raman spectrum of MoS_2_ NFs.

To rationalize these observations, we examined the physical properties of the nanoflowers. MoS_2_ NFs exhibit a porous morphology, with diameters ranging from 100 to 200 nm (Figure 2a–b). Detailed high‐resolution transmission electron microscopy (TEM) analyses of MoS_2_ nanoflowers have previously revealed expanded interlayer spacings of approximately 9.4–9.5 Å, attributed to molecular intercalation [25]. X‐ray diffraction revealed a significant shift of the characteristic (002) peak of MoS_2_ to a lower angle, indicating an expansion of the interlayer spacing from approximately 6.15 to 9.45 Å (9.35°), which indicates the successful increase in MoS_2_ interlayers spacing, leading to an improvement in the electronic structure and conductivity [25] (Figure 2c). Raman spectroscopy further indicated the presence of the characteristic J modes of 1T′ phases together with the A_1g_ and E^1^_2g_ peaks from the 2H phase [27] (Figure 2d). It was previously shown that bulk Mo 3d spectrum of 2H‐MoS_2_ exhibits peaks at 232.3 and 229.2 eV, assigned to the Mo^4+^ 3d_3/2_ and Mo^4+^ 3d_5/2_ components, respectively [25]. In contrast, the Mo 3d spectrum of our sample was fitted with five contributions (Figure S4), including dominant 1T′ MoS_2_ peaks at 231.5 and 228.3 eV, an S 2s signal near 226 eV, and 2H‐MoS_2_ components at 232.3 and 229.15 eV, which occur at nearly the same binding energies as those observed for bulk 2H‐MoS_2_. Similar results have also been reported in [25]. In addition, the increased intensity in the higher binding energy region, approximately 232–236 eV, indicates a significant contribution from Mo^6+^ species, suggesting enhanced surface oxidation. The formation of the 1T′ phase is attributed to the presence of the organic ligand and a possible electron transfer between the ligand and MoS_2_. Also, earlier studies found that the metallic phase works very efficiently for both EC and PEC processes [28, 29]. Taken together, our investigations suggest that the superior activity of the nanoflower morphology likely arises from the synergistic interplay between the high conductivity (1T′ phase), high density of exposed edges [28], and the nanoflower morphology, maximizing the specific surface area and the light absorption by GaAs/Si:p.

### 2.2 Morphological and Phase Effects

**FIGURE 3:**
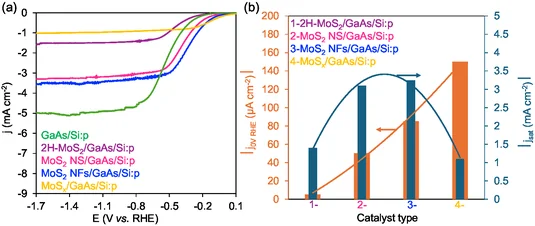
Impact of MoS_2_ morphology and phase on photocathode performance. (a) LSV curves showing the effect of morphology and phase of MoS_2_, on the electrochemical behavior of GaAs/Si:p photocathodes. (b) Photocurrent density at 0 V versus RHE and *j*_sat_ as a function of morphology and phase of MoS_2_.

To better understand the role of MoS_2_ cocatalysts morphology and their structural phase on the PEC performance, we compared MoS_2_ NFs with other benchmarked TDMs, namely MBA‐functionalized 1T′ MoS_2_ nanosheets (MoS_2_ NS) [27, 30], bulk 2H‐MoS_2,_ as well as amorphous MoS_*x*_. MoS_2_ NFs/GaAs/Si:p photocathodes showed the highest photocurrent and most positive onset potential, consistent with the EIS results (Figures 3a and S5). Bulk 2H‐MoS_2_ displays low onset potential and limited *j*_sat_, while MoS_2_ NFs offer balanced compromise between onset potential and photocurrent density (*j*_sat_) (Figures 3a, S5). This is also supported by the fact that MoS_2_ NFs and MoS_2_ NS display comparable trends in photocurrent and onset potential, underscoring the role of the 1T′ phase in governing PEC activity. Conversely, MoS_*x*_ exhibited a positive shift in *V*_onset_ and the highest photocurrent value at 0 V versus RHE (−150 µA cm^−2^), but its *j*_sat_ was the smallest one (Figure 3b).

We speculated that this limitation is attributed to the agglomerated structure of spray‐coated amorphous powder MoS_*x*_ and its lower crystallinity promoting low charge transfer across the semiconductor–catalyst–electrolyte interfaces. To confirm this hypothesis, we prepared optimized 30 min electrodeposited MoS_*x*_ films (Figure S6) and thereafter investigated the effect of thermal annealing at 400 °C. We demonstrate that the layered MoS_*x*_ films exhibit higher *j*_sat_ values with an acceptable *V*_onset_ compared to the MoS_*x*_ powder counterpart. However, thermal annealing at 400 °C further increases both *V*_onset_ and *j*_sat_ of the MoS_*x*_ films, confirming the synergistic importance of the preparation method, crystallinity, and morphology (Figure S7).

We corroborated the PEC results by spray depositing the TMD catalysts onto carbon paper and testing them in a three‐electrode electrochemical cell (EC). As shown in Figure S8a, MoS_2_ NFs displayed a greater current density in the EC configuration compared to sprayed MoS_*x*_ with almost a similar *V*_onset_. Nyquist plots revealed a significantly smaller semicircle for the MoS_2_ NFs electrode, indicating lower charge transfer resistance, *R*_ct_ (Figures S8b–d). The lowering in *R*_ct_ with increasing overpotential confirms improved HER kinetics, consistent with the enhanced saturation photocurrent observed in the PEC measurements.

**FIGURE 4:**
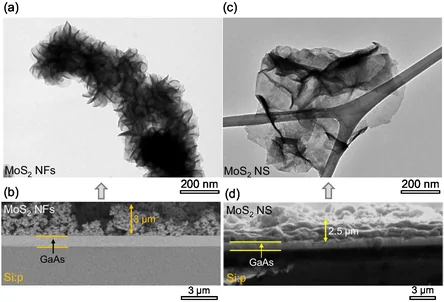
Morphological and structural comparison of MoS_2_ nanoflowers and nanosheets on GaAs/Si:p. TEM (a) and cross‐sectional SEM images (b) of MoS_2_ NFs/GaAs/Si:p. TEM (c) and cross‐sectional SEM images (d) of MoS_2_ NS/GaAs/Si:p.

We identified that nanosheets and nanoflowers shared similar PEC properties, albeit MoS_2_ NFs exhibiting higher *j*_sat_ values. To rationalize the differences between MoS_2_ NS and MoS_2_ NFs, cross‐sectional analyses under scanning electron microscopy (SEM) were conducted (Figure 4). The analyses confirmed that both electrode architectures possess comparable thicknesses. However, the nanoflower structure exhibits a significantly more porous morphology, which facilitates deeper light penetration [31]. SEM–EDS (energy‐dispersive X‐ray spectroscopy) revealed greater GaAs exposure beneath the nanoflowers, supporting the influence of morphology on PEC performance (Figure S9). Our investigations therefore suggest that the enhanced light diffusion likely accounts for the observed differences in saturation photocurrent density compared to the MoS_2_ NS phase.

Our findings underscore the critical role of the 1T′ phase and the nanoflower morphology of MoS_2_ in governing PEC performance. In contrast, electrodeposited MoS_*x*_ films can achieve high saturation current densities *j*_sat_ but exhibit a less favorable onset potential compared to TF‐MoS_2_‐400. We hypothesize that the improved crystallinity induced by annealing fosters better charge transport properties by establishing more ordered pathways for electron flow. This is supported by our EIS data, which revealed reduced charge transfer resistance as a function of annealing temperature (Figure S10). Previous studies from our group have shown that mild thermal annealing can significantly enhance the crystallinity of molybdenum sulfide [32].

**FIGURE 5:**
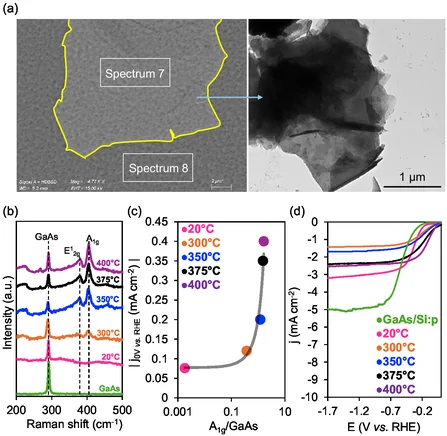
Effect of thermal treatment on the structural and PEC properties of TF‐MoS_2_. (a) SEM image showing the surface morphology of TF‐MoS_2_‐400 with the selected EDS regions (spectrum region 7 and 8 data are shown in Figure S12), and corresponding TEM image of the flake extracted from electrode surface. (b) Evolution of the Raman as a function of growth temperature. (c) Raman A_1g_/GaAs intensity ratio as a function of the current density at 0 V versus RHE for MoS_*x*_/GaAs/Si:p samples treated at different temperatures (300–400 °C), as extracted from the (d) LSV curves.

We then subjected the electrodeposited MoS_*x*_ films to thermal annealing at temperatures up to 400 °C. SEM–EDS images confirmed the uniform deposition of a MoS_2_ film on the GaAs/Si:p surface (Figure S11). After annealing, SEM–EDS and TEM analyses revealed that the rough texture of the GaAs/Si:p substrate remained observable through the MoS_2_ layer, confirming the formation of a TF, which likely enhances light absorption and, consequently, PEC performance (Figures 5a, S12). This process resulted in continuous improvement in film crystallinity, as evidenced by the enhanced 2H‐MoS_2_ Raman intensity (Figure 5b) compared to the amorphous peak first detected in the amorphous MoS_*x*_ Raman [33, 34]. This assignment was further confirmed by the X‐ray photoelectron spectroscopy survey spectrum (Figure S13), which showed that all characteristic elemental peaks were preserved after thermal treatment, together with the clear appearance of the Mo 3d and S 2p core‐level signals. Electrochemical performance characterization of the MoS_*x*_ electrodes annealed up to 400 °C revealed a continuous improvement in the *j*_sat_ and *V*_0V RHE_ with increasing temperature (Figures 5c–d). We show that both parameters reveal a clear correlation with crystallinity as a function of the change in A_1g_/GaAs Raman intensity (Figure 5c), while the effect on *V*_onset_ remains limited. These results underscore the critical role of crystallinity in electron transport.

### 2.3 Stability of Photocathodes

Our work highlights the influence of MoS_2_ morphology and phase composition on the PEC hydrogen evolution performance (Table S1). Therefore, to assess the resistance to morphological and structural degradation under prolonged electrochemical stress, we evaluated the stability of both molybdenum sulfide‐modified GaAs/Si:p photoelectrodes based on MoS_2_ NFs and TF‐MoS_2_‐400. The stability tests were performed under constant illumination at −0.3 V versus RHE in the presence of 0.2M H_2_SO_4_ (Figure S14). Both electrodes show stable current variation for 120 min. The initial photocurrent decrease is attributed to the dark‐to‐light transition, while the subsequent increase likely results from gradual heating under illumination.

**FIGURE 6:**
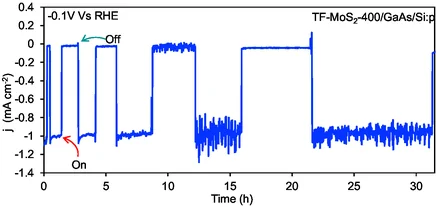
Long‐term stability of the optimized TF‐MoS_2_‐400 electrode.

TEM and SEM analyses (Figure S15) revealed no significant post‐electrolysis alterations on the surface of the MoS_2_ NFs/GaAs/Si:p, confirming the preservation of its morphology. To further evaluate changes in the cocatalysts structural composition, Raman spectroscopy was employed (Figure S16). The Raman analysis revealed no notable shifts in peak positions or intensities, confirming the structural stability of both photocathodes. To verify that HER is the primary reaction occurring on the electrode, we conducted a 75‐min test at −0.3 V versus RHE under light irradiation and analyzed the gaseous products using gas chromatography. Under these conditions, we determined a ~97% Faradaic efficiency for H_2_, indicating that the photocurrent is predominantly generated by the HER process, with negligible contribution from any undesired reduction of the catalyst itself. In addition, the long‐term stability of the optimized TF‐MoS_2_‐400 electrode was evaluated by measuring its transient photocurrent response under repeated light on/off cycles over 30 h of operation. The results confirm the excellent stability of the optimized electrode (Figure 6).

## 3 Conclusion

In this study, we systematically explored the interplay between morphology, crystallinity, and structural phase of molybdenum sulfide catalysts to optimize their performance in PEC H_2_ production. We developed a hybrid photoelectrode architecture, integrating 1‐μm‐thick GaAs layers, grown epitaxially on a low‐cost p‐doped Si substrate with MoS_2_ nanostructures. Our investigations revealed that the crystalline MoS_2_ nanoflower morphology with 1T′ domains achieves an optimal balance between electron conductivity and optical response. This unique structure resulted in superior photocurrent density and a high *V*_onset_. Furthermore, we demonstrated the critical role of crystallinity by examining the effect of thermal annealing on electrodeposited MoS_*x*_. A clear positive correlation was observed between enhanced crystallinity and improved photocatalytic performance. Notably, both MoS_2_ NFs and TF‐MoS_2_‐400 cocatalysts achieved a high Faradaic efficiency of approximately 97% for HER when loaded on the GaAs/Si:p photocathode, confirming the selectivity and robustness of the hybrid architecture. Overall, the synergistic integration of the GaAs/Si:p junction with engineered MoS_2_ nanostructures establishes an efficient and sustainable platform for PEC hydrogen production. Our findings not only advance our understanding of the structure–property–performance relationship in PEC systems but also pave the way for the development of scalable, platinum group metal‐free photoelectrodes for practical solar fuel applications.

## Author Contributions

Conceived the idea and designed the experiments – Z.A. and D.V., implemented the experimental setup and conducted the experiments ‍– Z.A. and F.M., methodology – all authors, resources – B.F., G.L., Y.L., N.B., C.C., C.S., and D.V., funding acquisition – D.V. and C.C., project administration ‍– C.S. and D.V., supervision – D.V., writing – original draft ‍– Z.A., writing ‍– review and editing – all authors. All authors discussed and revised the manuscript.

## Funding

This work was supported by the Agence Nationale de la Recherche (ANR‐22‐PEHY‐0013 and ANR‐21‐ESRE‐0012).

## Conflicts of Interest

The authors declare no conflicts of interest.

## Supporting information

Supplementary Material

## Data Availability

The data that support the findings of this study are available from the corresponding author upon reasonable request.
