# Supplementary material for: The Role of Morphology and Phase Engineering in Molybdenum Sulfide for Robust Photoelectrochemical Hydrogen Evolution on III–V Photoelectrodes
Source: ChemSusChem. 2026 Sep 26;19(19):e71110. doi: 10.1002/cssc.71110 (PMC13615551; doi:10.1002/cssc.71110)
Supplement: Supplementary file 1 [file CSSC-19-e71110-s001.pdf]

The Role of Morphology and Phase Engineering in Molybdenum Sulfide for Robust Photoelectrochemical Hydrogen Evolution on III-V photoelectrodes

Zakaria Anfar[1,*], Fatima Merhi[2], Bruno Fabre[3], Gabriel Loget[2], Yoan Léger[4], Nicolas Bertru[4], Charles Cornet[4], Chrystelle Salameh[1] and Damien Voiry[1,*]

[1] Institut Européen des Membranes (IEM), UMR 5635, CNRS, Université de Montpellier, ENSCM, 34080 Montpellier, France
E-mail: *corresponding author : zakaria.anfar@umontpellier.fr; damien.voiry@umontpellier.fr

[2] Institut des Sciences Moléculaires (ISM), UMR 5255, CNRS, Université de Bordeaux, Bordeaux INP, 33607 Pessac, France
[3] Institut des Sciences Chimiques de Rennes (ISCR), UMR 6226, CNRS, Université de Rennes, 35000 Rennes, France
[4] Institut FOTON, UMR 6082, CNRS, Université de Rennes, INSA Rennes, 35000 Rennes, France

Reagents and materials

Ammonium tetrathiomolybdate $(NH_4)_2[MoS_4]$, sulfuric acid (H_2SO_4, 99.99%), isopropyl alcohol (ACS reagent), MoS_2 powder, *n*-butyllithium, hexane, filter paper and (R)-(+)-α-methylbenzylamine (MBA) were purchased from Sigma-Aldrich. All the reagents were used as received without further purification. Nafion™ D521CS perfluorinated solution (ion power, 5% weight). Carbon paper (CP, Sigracet 39 AA Brand: SGL Carbon) was obtained from Fuel Cell Store. A GaAs:p wafer (Freiberger Compound Materials GmbH) was used for some characterization measurements. All aqueous solutions were prepared using Ultra-pure water.

Molecular beam epitaxy of GaAs on Si:p

After a last-HF chemical etching of the Si wafer native oxide, the substrate was introduced in the solid source molecular beam epitaxy chamber. Hydrogen desorption was performed by heating the substrate at 800°C for 10 minutes, and the temperature was ramped down to 550°C, where elemental Ga and As atoms were supplied in the ultra-high vacuum chamber from solid source cells, with a V/III Beam Equivalent Pressure ratio of 2. After 1 µm-thick deposition, the sample was cooled down to room temperature under As pressure.

Catalysts preparation

MoS_2 nanoflowers intercalated MBA were synthesized *via* a hydrothermal method as reported before[1]. A solution of $(NH_4)_2[MoS_4]$ (66 mg in 20 mL H_2O) was sonicated for 10 min, followed by the addition of MBA (32.3 µL) and HCl (37 vol%, 10 µL). The mixture was heated at 200 °C for 12 h in a Teflon-lined autoclave. The black product was filtered, washed with water and acetone, and vacuum-dried at room temperature. MoS_x was synthesized via acidification of $(NH_4)_2[MoS_4]$. A 10 mM aqueous solution of $(NH_4)_2[MoS_4]$ was first sonicated, then maintained under an argon atmosphere with stirring for 25 minutes, after which 0.05 mL of concentrated H_2SO_4 was added. The MoS_2 bulk (2H MoS_2) powder was purchased from Alfa Aesar and used without modification. Single-layer MoS_2 was obtained by lithium intercalation of bulk MoS_2 (400 mg) with *n*-butyllithium (4 mL) in hexane at 65 °C for 48 h under argon as described before [2,3]. After filtration, washing, and water exfoliation with sonication, nanosheets were isolated by centrifugation. For functionalization, DMSO was added, and MoS_2 reacted with MBA molecule (10× excess) for 48 h, yielding functionalized MoS_2 solutions.

Carbon paper-based electrodes

Catalyst inks were prepared by dispersing 200 mg of catalyst and 30 mg of Vulcan carbon in a mixture of 9 mL IPA, 1 mL H_2O, and 50 µL Nafion. After 30 min of sonication (20°C), the ink was spray-coated onto carbon paper (CP), achieving a loading of 1 mg cm^{-2}.

GaAs/Si:p based photoelectrodes using spray deposition

Catalyst inks were prepared by dispersing the desired amount of catalyst in a solvent mixture of 1.5 mL deionized water and 4.5 mL isopropanol (IPA), to achieve a final concentration of 0.5 mg mL^{-1}. Then, 1 wt. % of Nafion ionomer was added as a binder. The mixture was stirred for 30 min to ensure homogeneity. The ink was then spray-coated onto GaAs/Si:p substrates maintained at 100 °C. The resulting catalyst loadings, determined from mass differences before and after deposition, yielded an average loading of 0.33 mg cm^{-2}.

GaAs/Si:p based photoelectrodes using electrodeposition

GaAs/Si:p photocathodes were immersed in 1 M Na_2SO_4 aqueous solution containing 1 mM $(NH_4)_2[MoS_4]$. In a three-electrode system, GaAs/Si:p was used as the working electrode (WE), Pt foil as the counter-electrode (CE), and Ag/AgCl (3M NaCl) as the reference electrode (RE) to perform the deposition by chronoamperometry (CA). A fixed potential of -1.2 V *vs.* Ag/AgCl under illumination (100 mW/cm^2) was applied for 30 min during the PEC deposition of MoS_x. After CA, the electrodes were rinsed with water and finally dried under N_2. The electrodeposited MoS_x films were thermally annealed for 2 h at temperatures ranging from 300 to 400 °C under a continuous Ar/H_2 flow of 100 sccm.

Photoelectrodes contact engineering

The electrode backside was scratched to remove surface oxide and improve electrical contact. An InGa droplet was applied, and a metal wire was inserted to form an ohmic connection. Silver paste was added and cured for 15 minutes, then covered with epoxy for mechanical and chemical protection. The electrode edges were also sealed with epoxy to prevent electrolyte leakage during PEC measurements.

Characterization techniques

Transmission electron microscopy (TEM) was performed using a JEOL 1400 Flash microscope. X-ray photoelectron spectroscopy (XPS) analyses were conducted on an ESCALAB 250 system (Thermo Electron) using a monochromatic Al Kα line (1486.6 eV) as the excitation source. X-ray diffraction (XRD) patterns were obtained using an X'Pert Powder diffractometer. Scanning electron microscopy (SEM) was conducted on a Hitachi S4800, while SEM-EDS analysis was performed using a Zeiss EVO HD15 microscope equipped with an Oxford Instruments X-Max N SDD EDS detector. Raman spectra (RS) were recorded using a Renishaw inVia Raman microscope with a 532 nm excitation wavelength and a ×50 objective lens.

Experimental setup

Electrochemical measurements were performed with a PARSTAT MC Multichannel Potentiostat (PMC-200, AMETEK Princeton Applied Research) in 0.2 M H_2SO_4 using a three-electrode PEC cell. The GaAs/Si:p photoelectrode was illuminated under AM 1.5G light at 100 mW cm^{-2}, with graphite and Ag/AgCl (sat. KCl) as the counter and reference electrodes,

respectively. LSV and CV measurements were recorded at a fixed scan rate of 10 mV s^{-1}, and current densities were normalized to the exposed electrode area. Potentials were converted to the RHE scale using:

E(RHE) = E(Vs. Ag/AgCl) + 0.059pH + 0.197V

The long-term stability of the photoelectrode was evaluated via chronoamperometry (*j*-*t*) measurements under continuous illumination. Additionally, electrochemical impedance spectroscopy (EIS) was performed in the frequency range of 100 kHz to 0.01 Hz using a small AC amplitude at a constant applied potential to analyze charge transfer resistance and interfacial behaviour. To estimate the Faradaic efficiency of hydrogen evolution, gas chromatography (GC) was employed to analyze the amount of H_2 generated in a well-sealed PEC cell. The measurements were conducted under a constant applied potential using chronoamperometry (*j*–*t*), ensuring a steady charge passed over time. The volume of H_2 collected in the headspace was quantified and compared to the theoretical amount expected from the total charge passed, allowing precise calculation of the Faradaic efficiency.

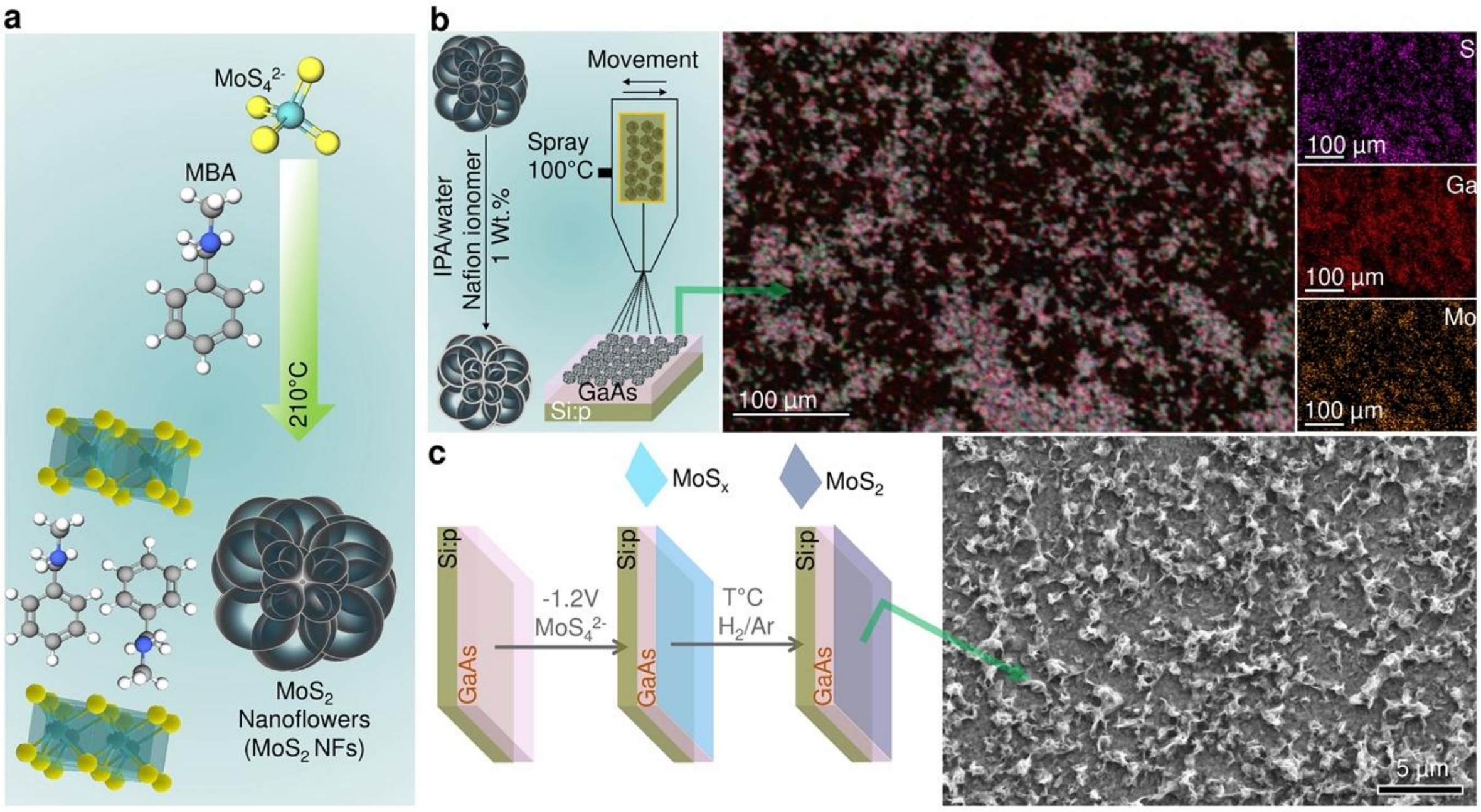

Figure S1. (a) Hydrothermal method for the preparation of MoS_2 nanoflowers (MoS_2 NFs), **(b)** scheme presenting the spray technique for MoS_2 NFs coupled with SEM mapping showing the exposed GaAs surface of MoS_2 NFs/GaAs/Si:p and **(c)** scheme presenting the electrodeposition assisted thermal reduction process for the preparation of MoS_2 thin films coupled with SEM image.

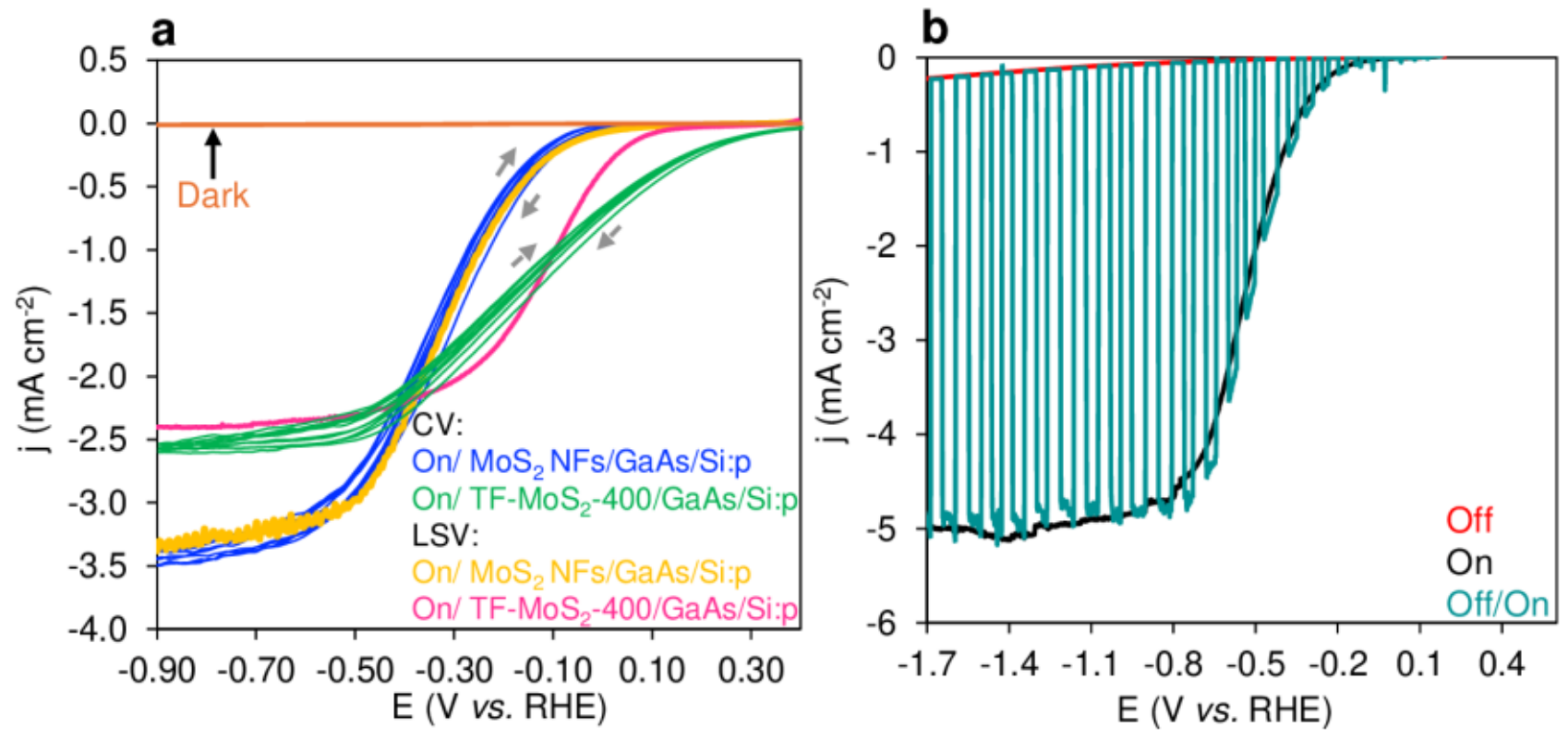

Figure S2. (a) Cyclic voltammetry (CV) and LSV of MoS_2 NFs/GaAs/Si:p, and TF-MoS_2-400/GaAs/Si:p under simulated solar illumination and **(b)** Linear sweep voltammetry (LSV) curves of bare GaAs/Si:p under off/on illumination.

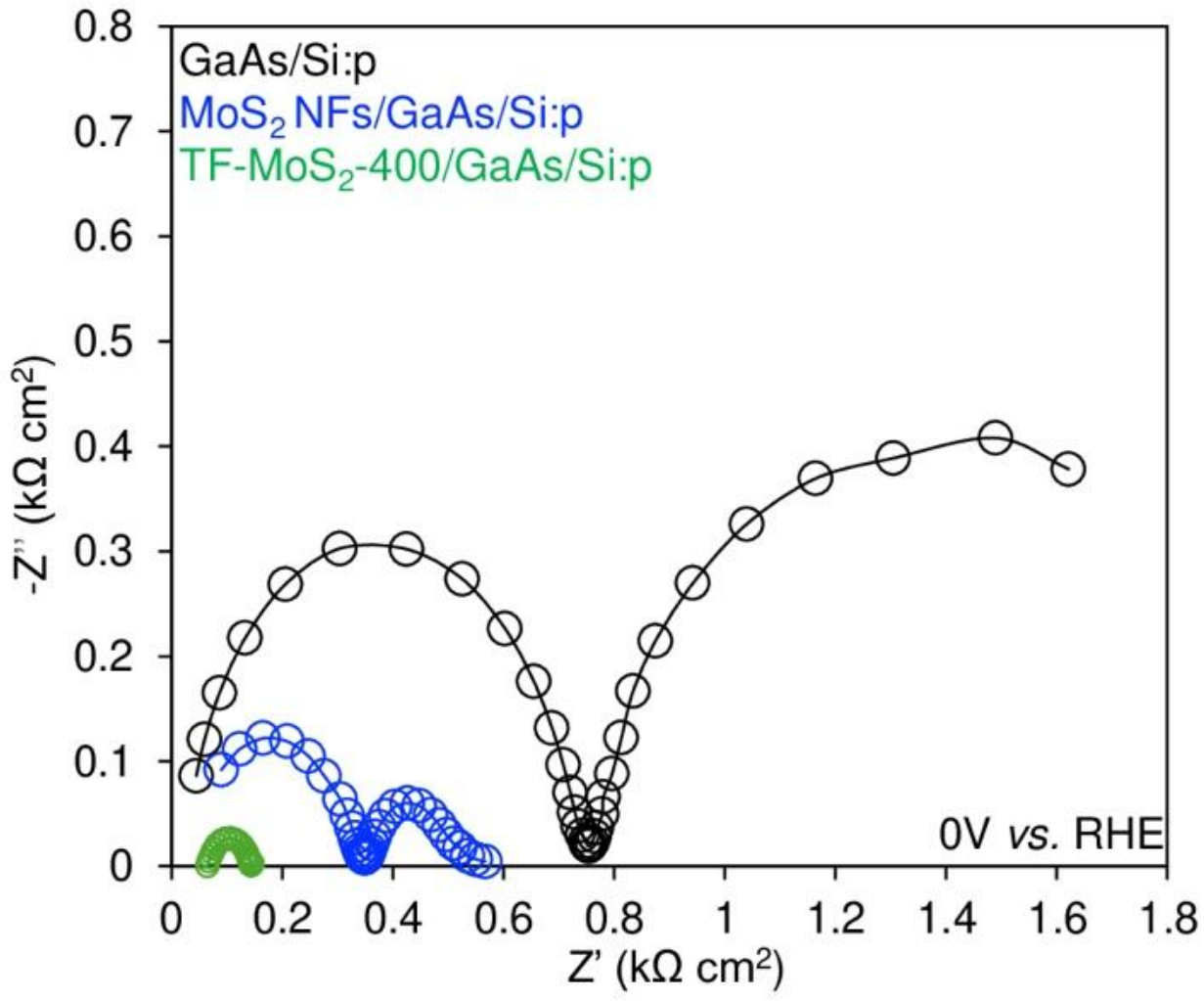

Figure S3. Nyquist plots from electrochemical impedance spectroscopy (EIS) at 0 V vs RHE of different photocathodes under simulated solar illumination.

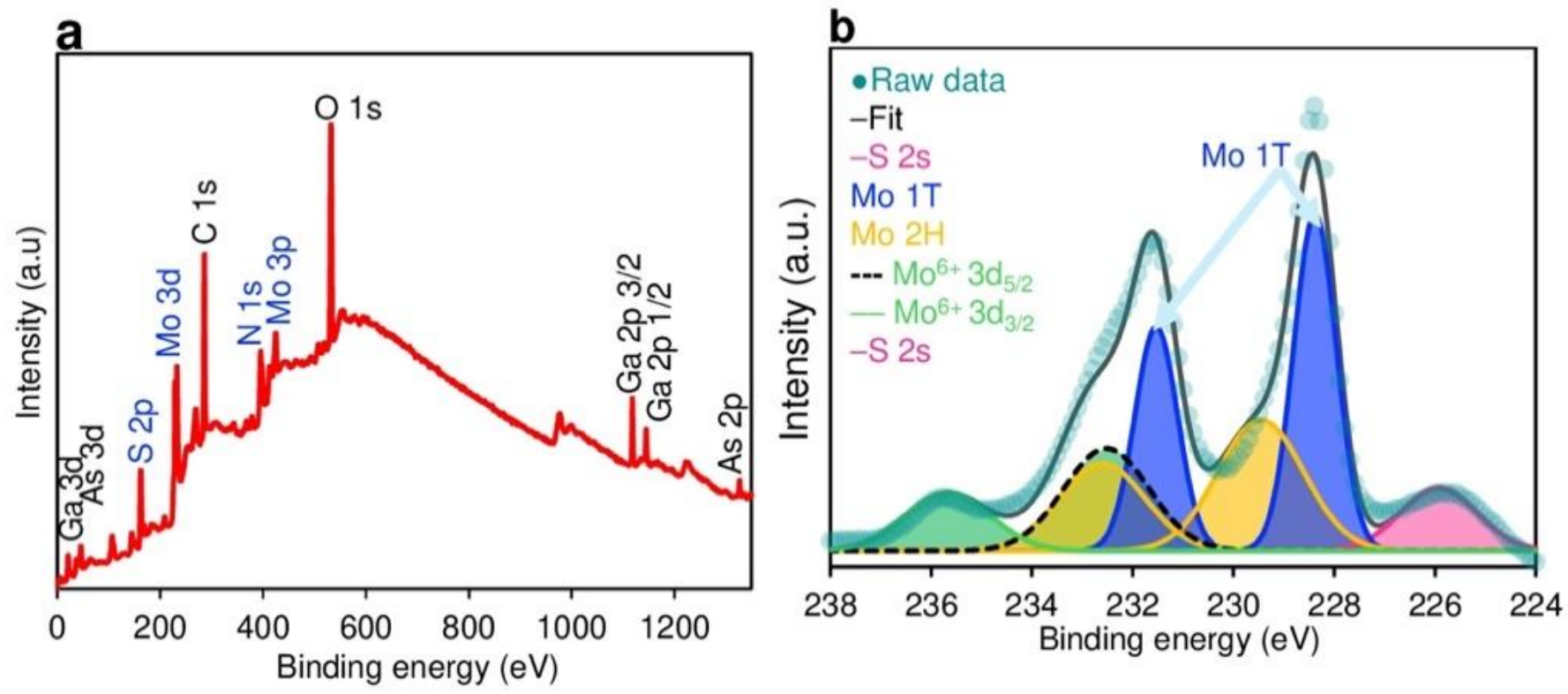

Figure S4. **(a)** XPS survey spectrum and **(b)** Mo 3d High-resolution XPS spectrum of MoS_2 NFs/GaAs

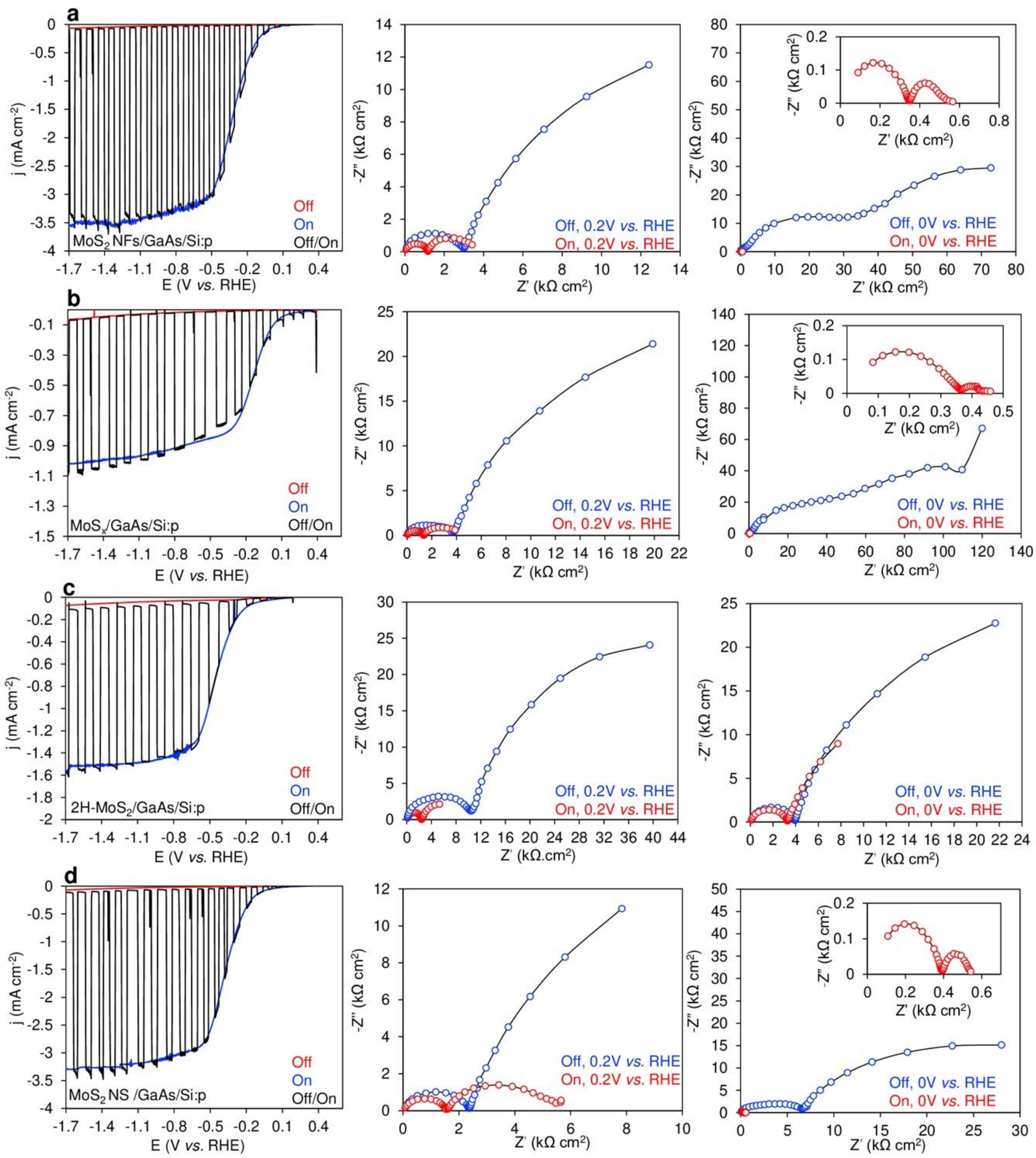

Figure S5. LSV curves and Nyquist plots at 0.2 V and 0 V vs RHE of photoelectrodes under off/on illumination of **(a)** MoS_2 NFs/GaAs/Si:p**, (b)** MoS_x/GaAs/Si:p**, (c)** 2H-MoS_2/GaAs/Si:p and **(d)** MoS_2 NS/GaAs/Si:p.

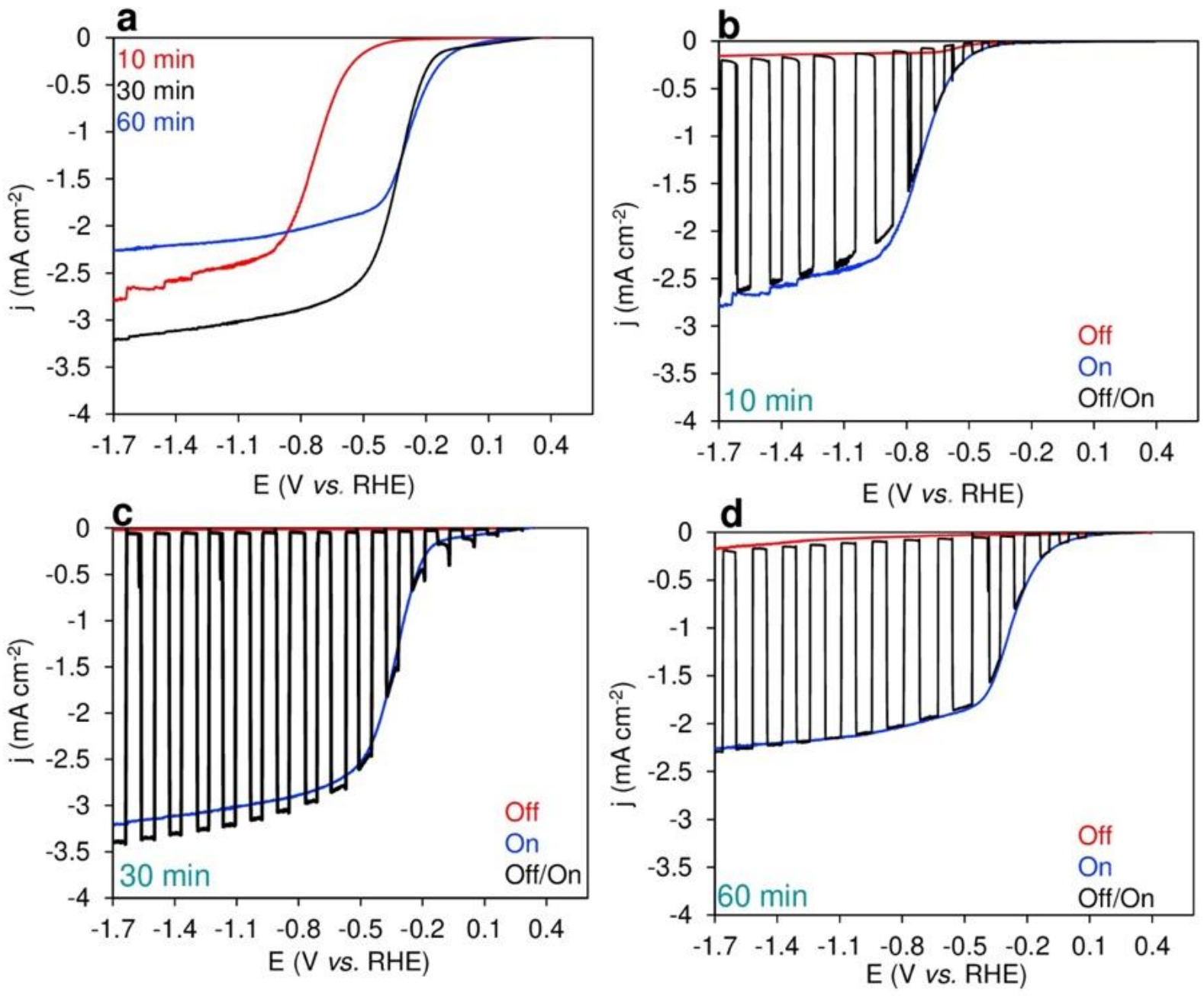

Figure S6. (a) LSV curves comparing the effect of electrodeposition time and dark/light LSV of **(b)** 10 min**, (c)** 30 min and **(d)** 60 min of MoS_x electrodeposited onto GaAs/Si:p.

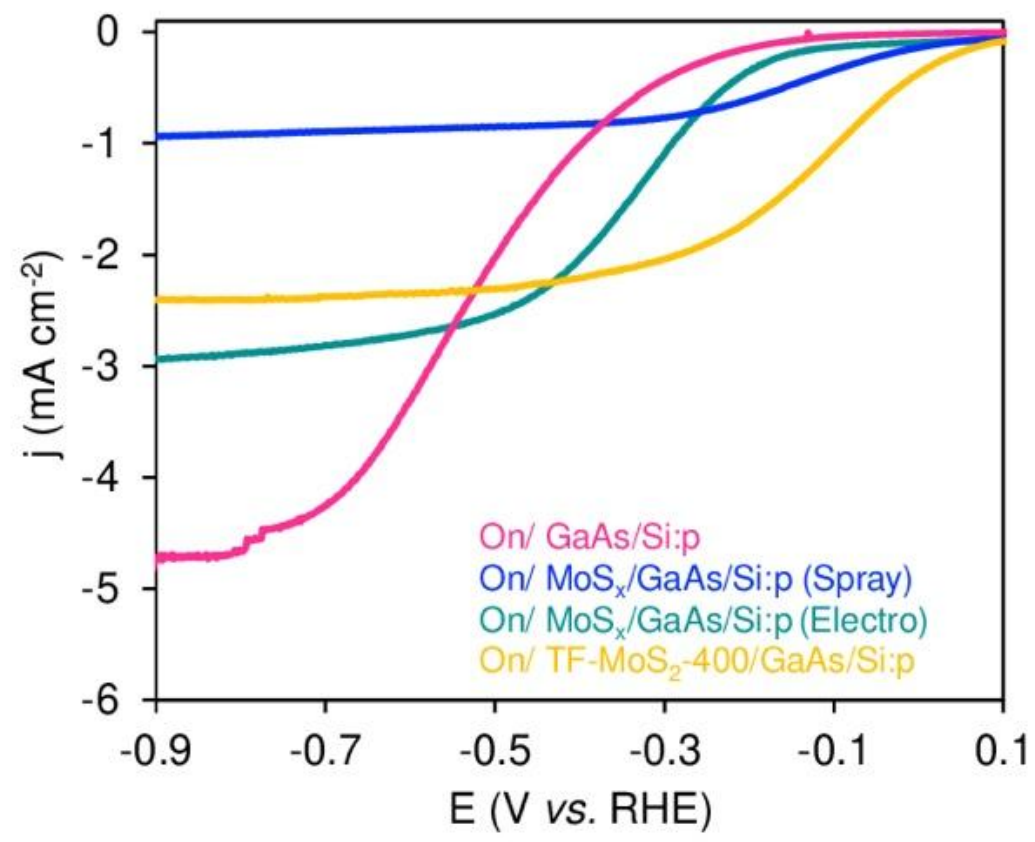

Figure S7. LSV curves under illumination comparing different MoS_x catalysts deposited on GaAs/Si:p.

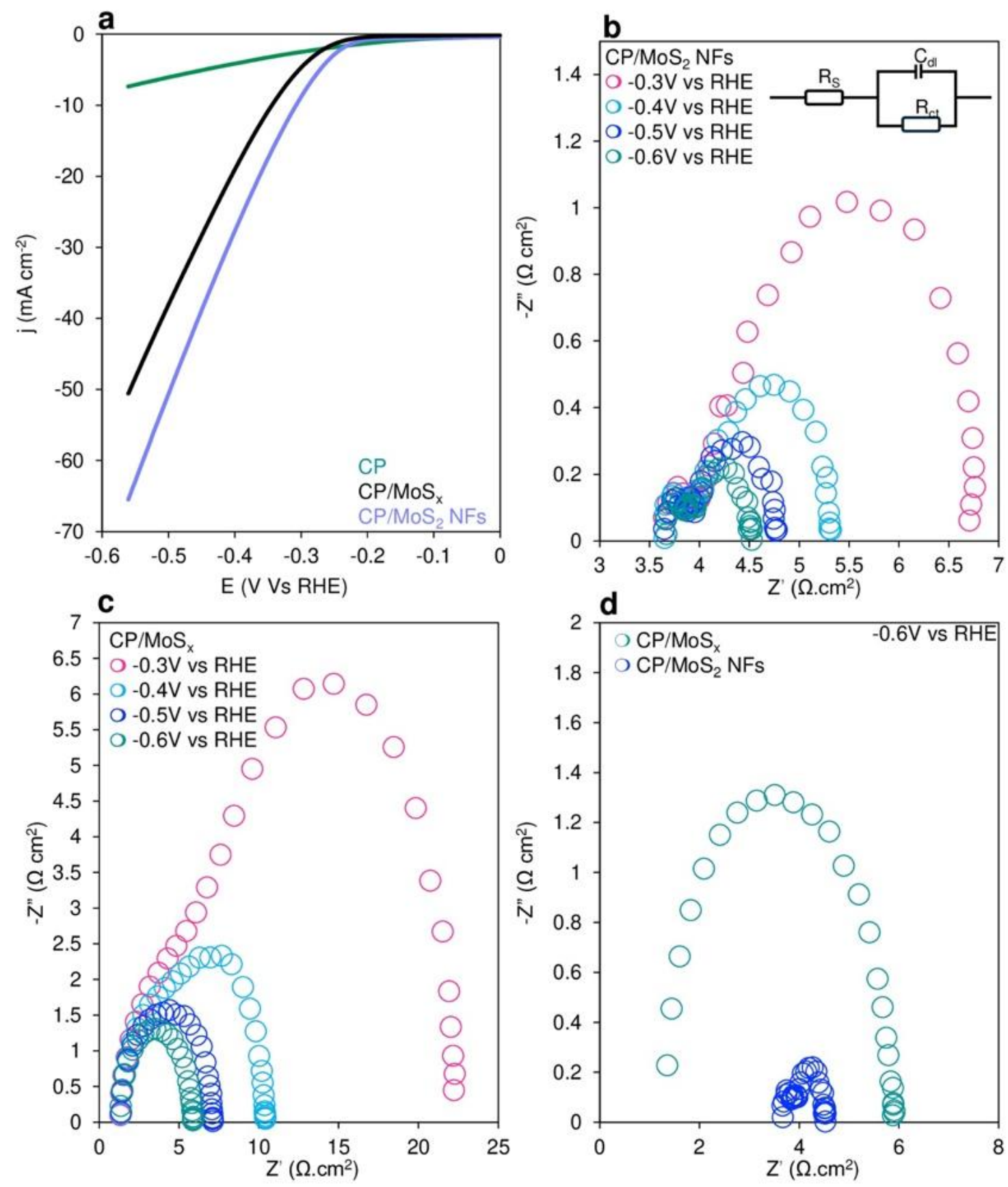

Figure S8. (a) LSV curves comparing the HER efficiencies in three electrodes configuration of spray powder MoS_2 NFs and MoS_x into carbon paper (CP) **(b)** Nyquist plots of MoS_2 NFs under different potentials, **(c)** Nyquist plots of MoS_x under different potentials, **(d)** Nyquist plots comparing MoS_2 NFs and MoS_x at -0.6V vs RHE.

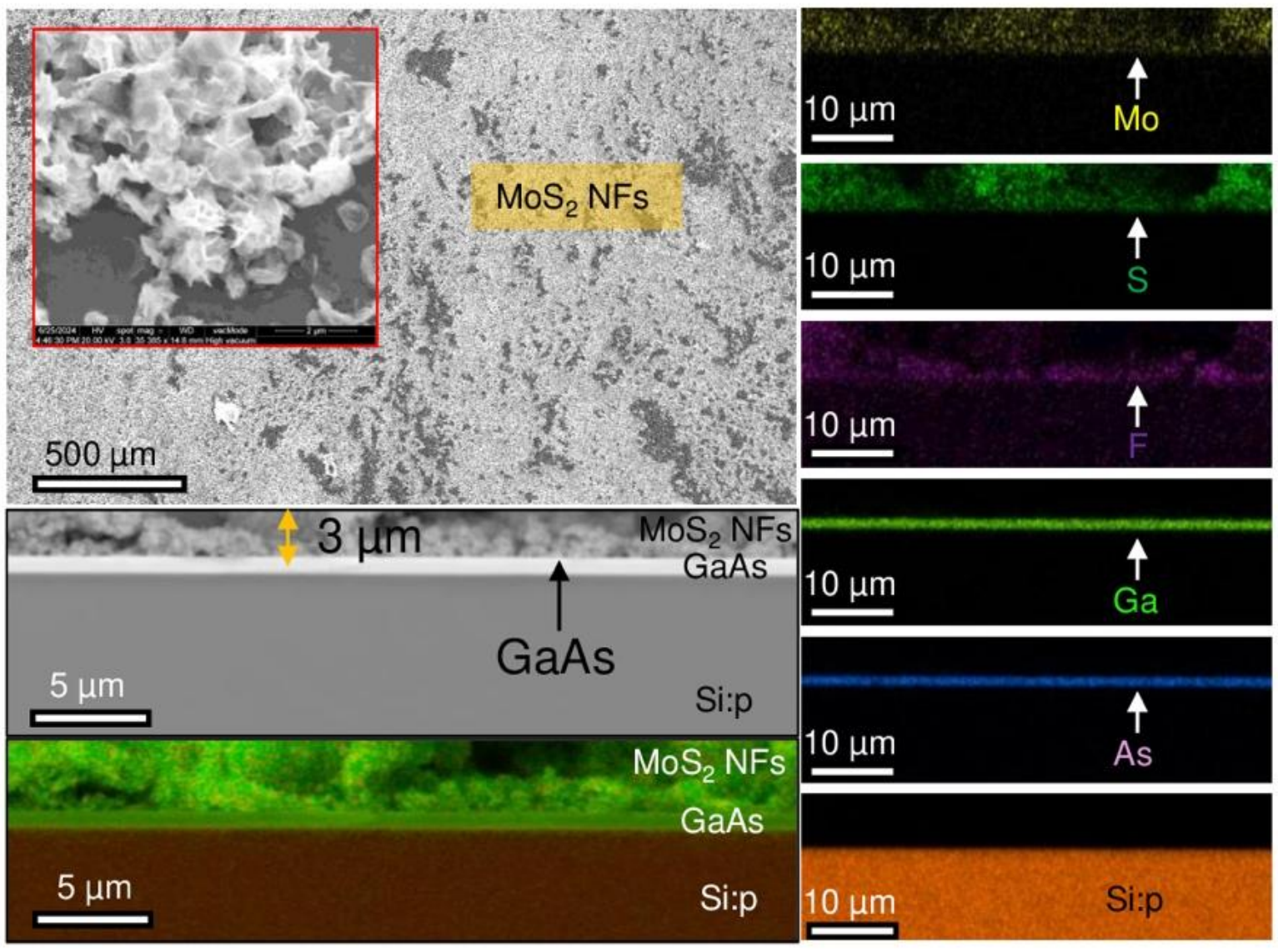

Figure S9. Cross-section SEM mapping of MoS_2 NFs/GaAs/Si:p.

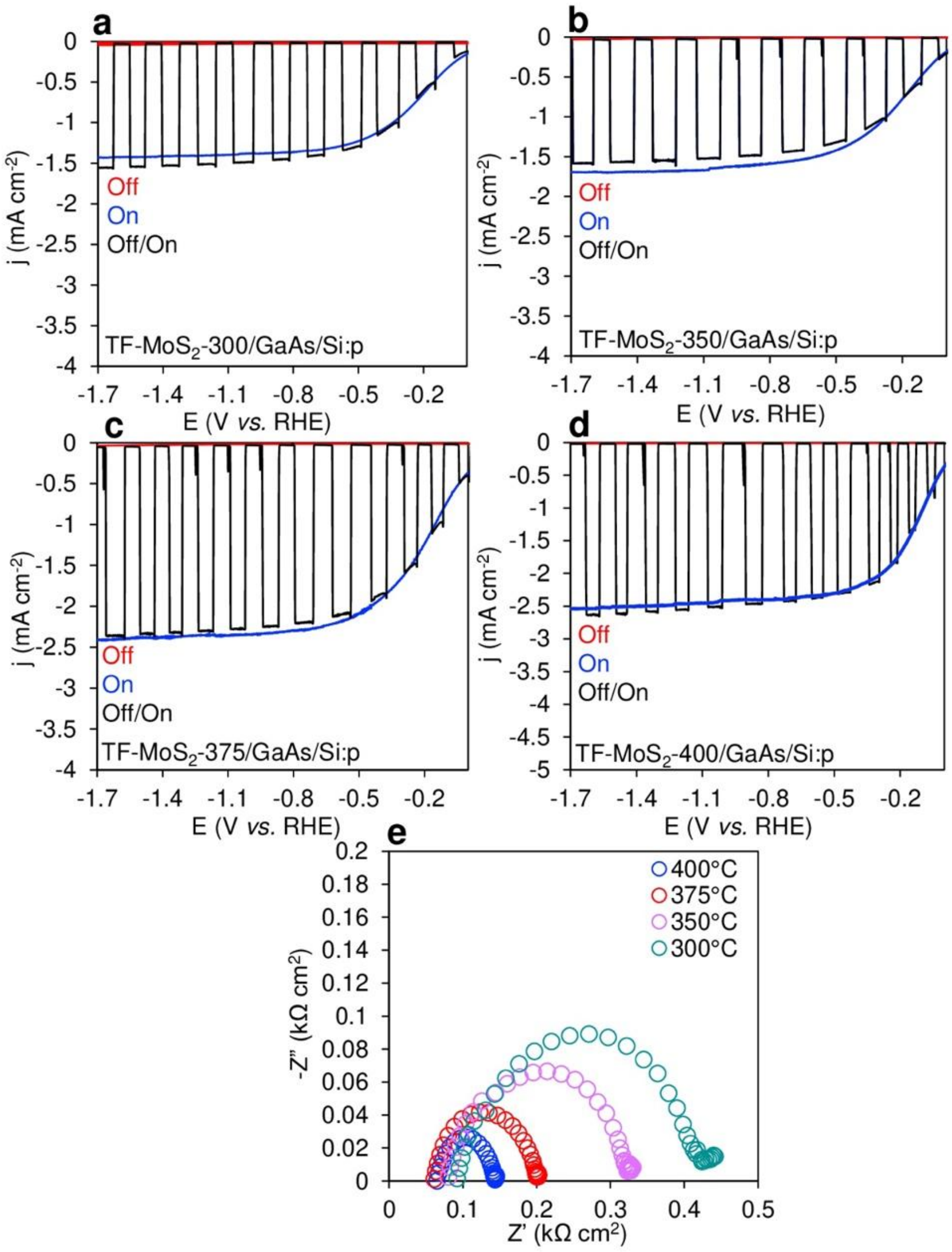

Figure S10. (a), (b), (c) and **(d)** LSV curves under on/off illumination cycles comparing the temperature effect of MoS_x electrodeposited onto GaAs/Si:p, **(e)** Nyquist plots comparing the temperature effect measured at 0 V *vs.* RHE.

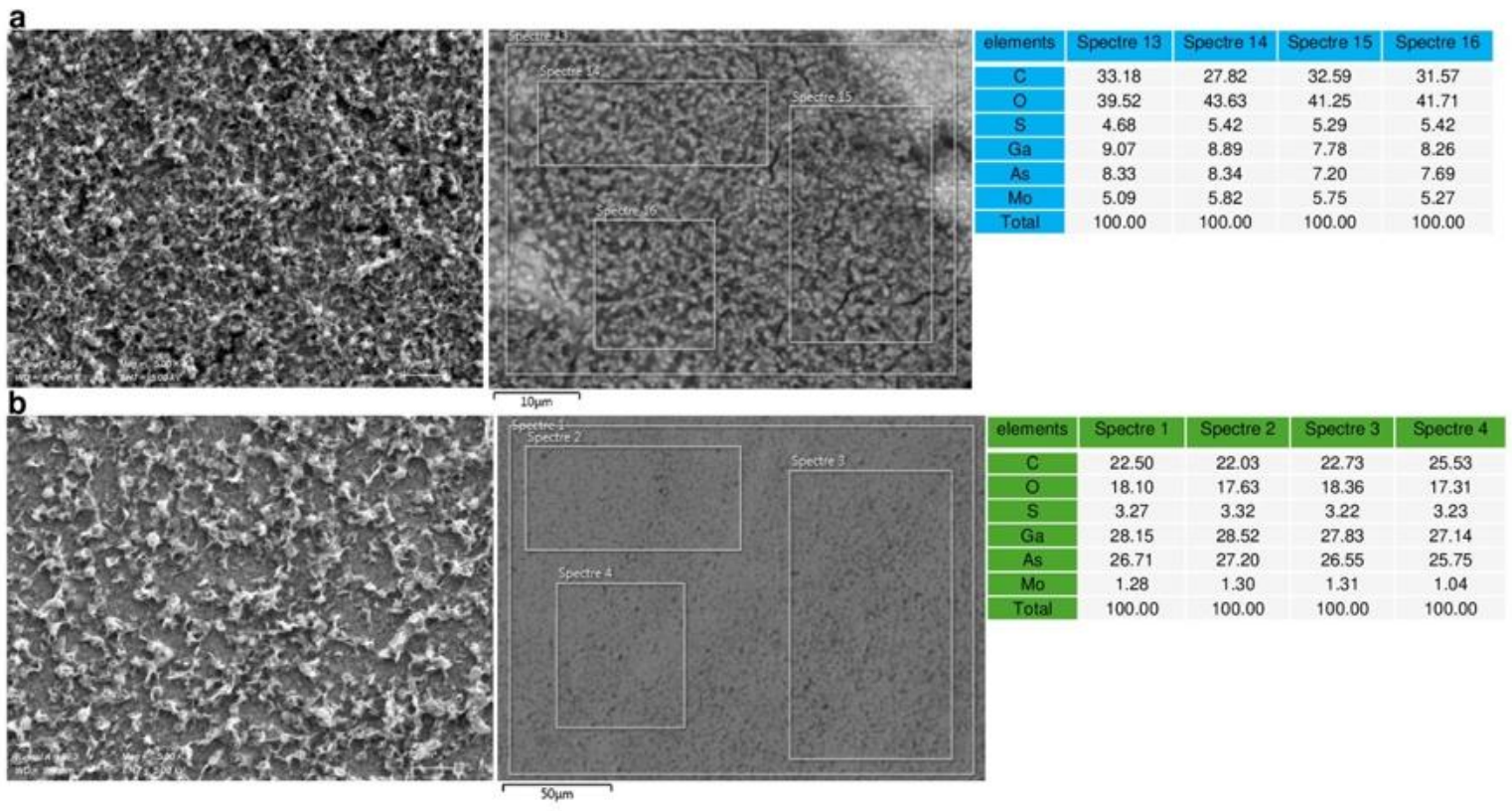

elements	Spectre 13	Spectre 14	Spectre 15	Spectre 16
C	33.18	27.82	32.59	31.57
O	39.52	43.63	41.25	41.71
S	4.68	5.42	5.29	5.42
Ga	9.07	8.89	7.78	8.26
As	8.33	8.34	7.20	7.69
Mo	5.09	5.82	5.75	5.27
Total	100.00	100.00	100.00	100.00

elements	Spectre 1	Spectre 2	Spectre 3	Spectre 4
C	22.50	22.03	22.73	25.53
O	18.10	17.63	18.36	17.31
S	3.27	3.32	3.22	3.23
Ga	28.15	28.52	27.83	27.14
As	26.71	27.20	26.55	25.75
Mo	1.28	1.30	1.31	1.04
Total	100.00	100.00	100.00	100.00

Figure S11. SEM-EDS image showing the surface morphology of **(a)** MoS_x/GaAs (prepared by electrodeposition and before thermal treatment) and **(b)** TF-MoS_2-400/GaAs (after thermal treatment at 400°C).

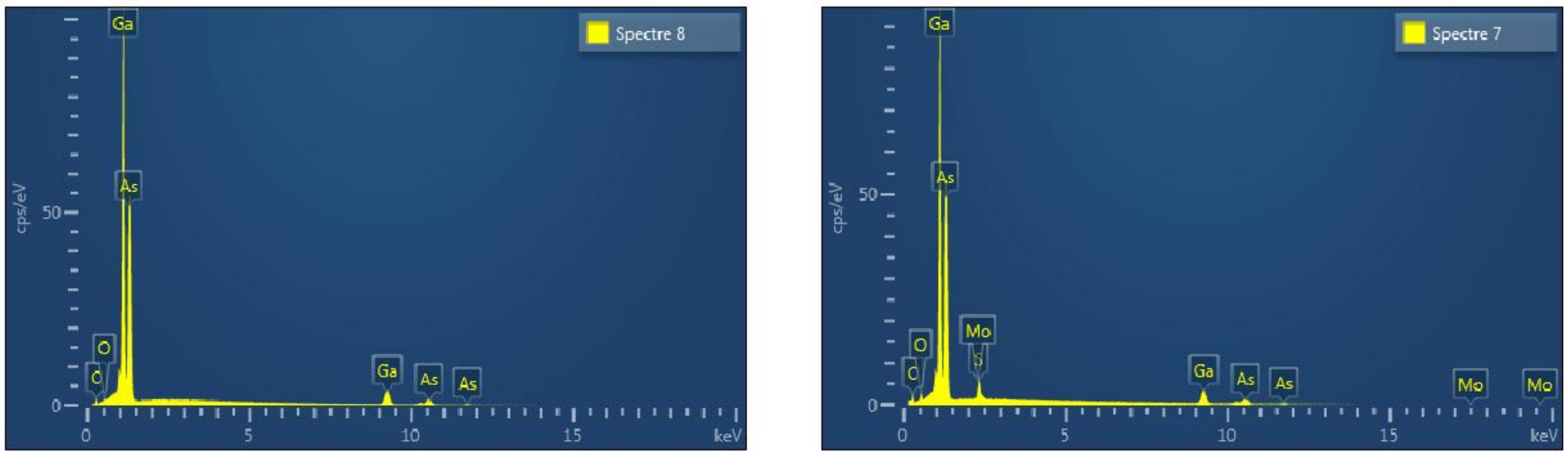

Figure S12. SEM-EDS of selected area of TF-MoS_2-400/GaAs of **Figure 5a**

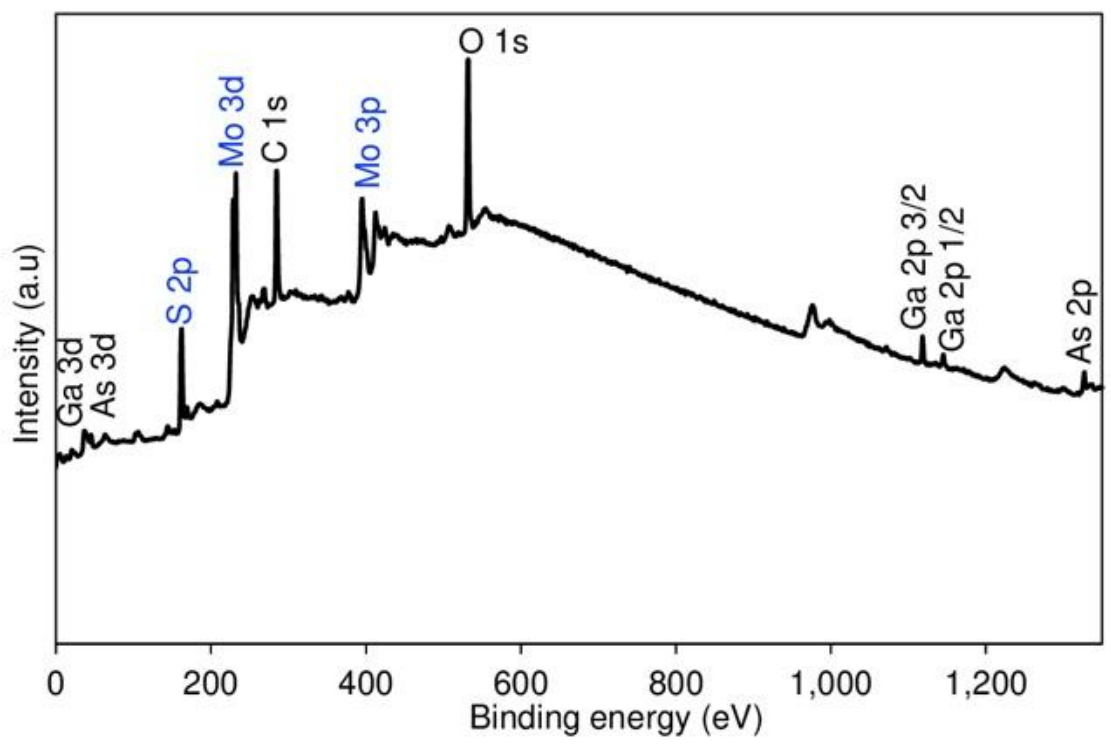

Figure S13. XPS survey spectrum of TF-MoS_2-400/GaAs

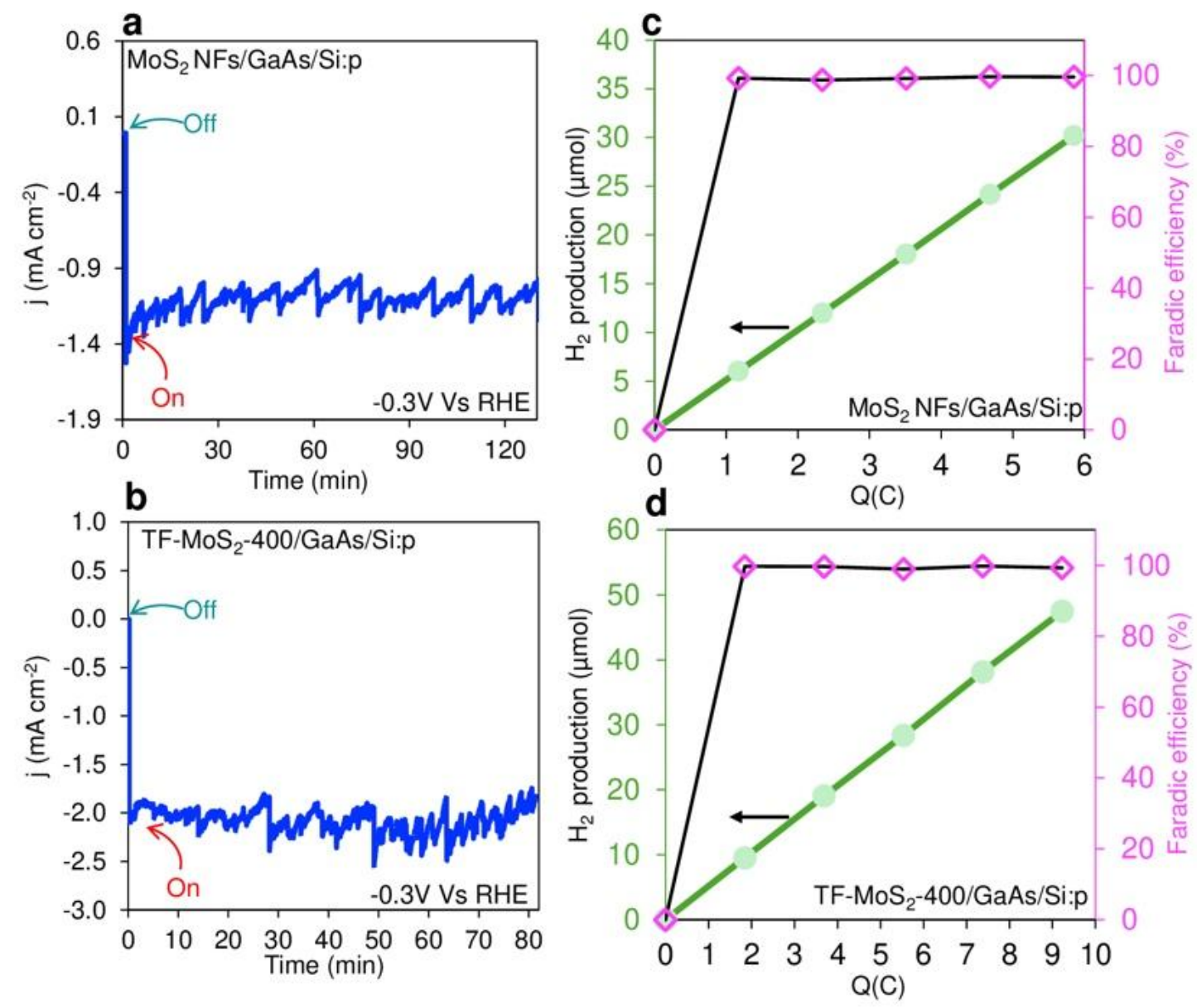

Figure S14. Chronoamperometry curves of **(a)** MoS_2 NFs/GaAs/Si:p and **(b)** TF-MoS_2-400/GaAs/Si:p at -0.3 V vs RHE under light illumination. H_2 evolution and faradaic efficiency measured by GC at -0.3 V vs RHE for: **(c)** MoS_2 NFs/GaAs/Si:p and **(d)** TF-MoS_2-400/GaAs/Si:p.

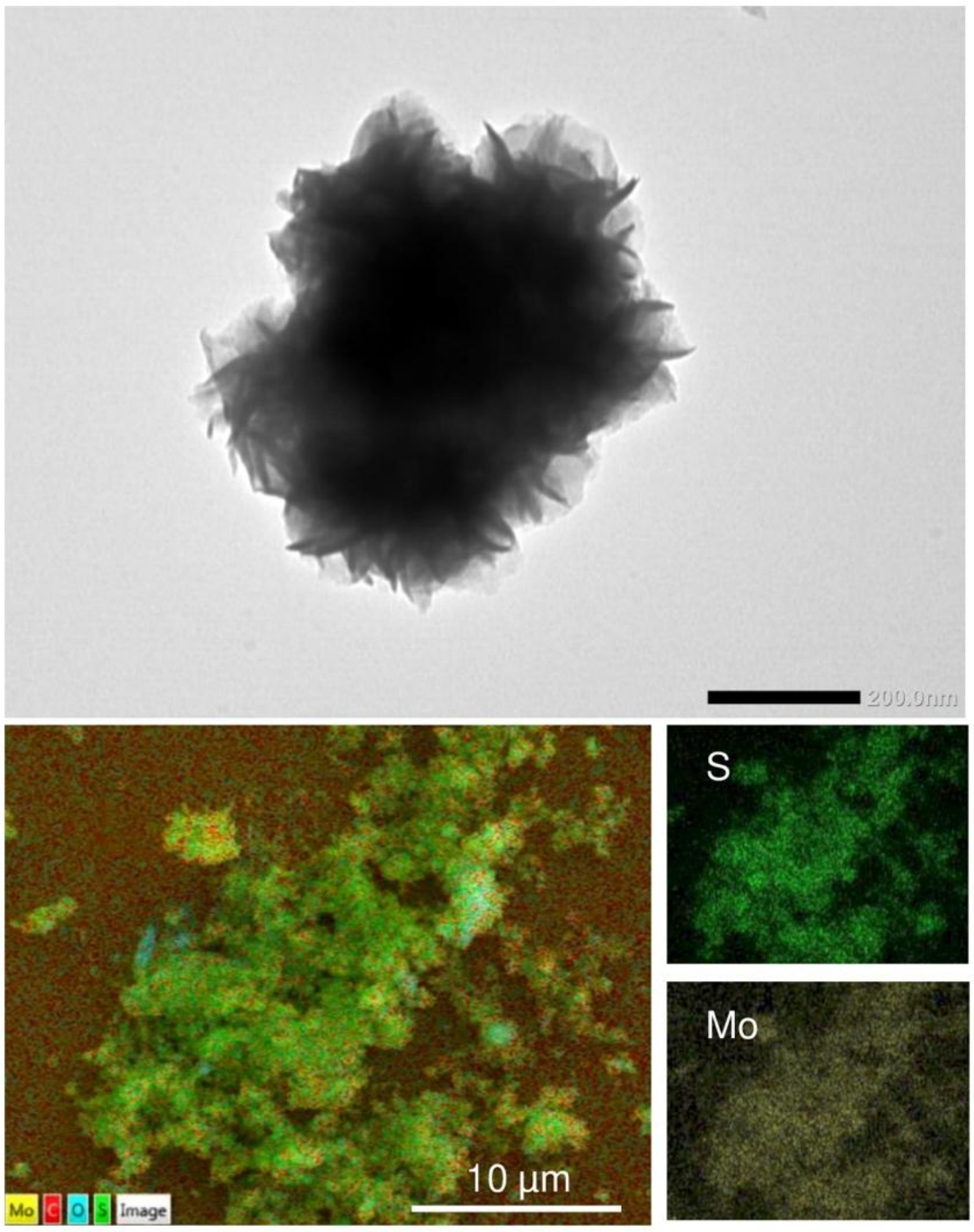

Figure S15. TEM (top) and SEM-EDS (bottom) images showing the morphology of MoS_2 NFs after the HER stability test.

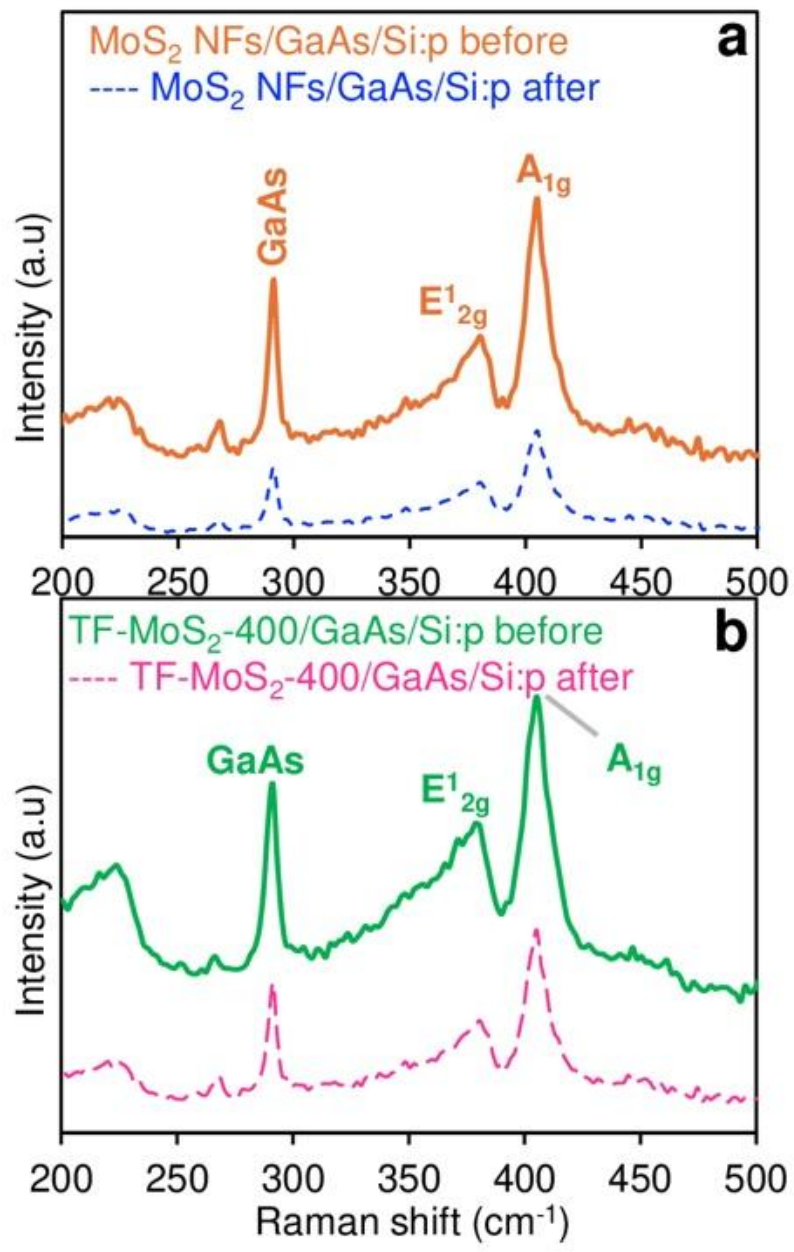

Figure S16. Raman spectra before and after HER stability test of **(a)** MoS_2 NFs/GaAs/Si:p and (**b**) TF-MoS_2-400/GaAs/Si:p.

Table S1. Quantitative comparison of key parameters highlights the influence of MoS_2 morphology.

Catalyst	Morphology	Electrolyte	V_{onset} vs RHE at -0.5 mA cm^{-2}	j_{sat} (mA cm^{-2})
MoS_2	1T ‘Nanosheets	0.2 M H_2SO_4	-0.22 V	-3.31
MoS_2	Bulk 2H		-0.4 V	-1.49
MoS_2	Nanoflowers		-0.17 V	-3.51
MoS_x	Amorphous		-0.16 V	-1
MoS_x	Amorphous (Edep)		-0.22 V	-3.2
MoS_2	Crystalline (Edep + 400 °C)		-0.025 V	-2.5

Edep = Electrodeposition method

References

[1] Z. Bian, K. Kato, T. Ogoshi, Z. Cui, B. Sa, Y. Tsutsui, S. Seki, M. Suda, “Hybrid Chiral MoS 2 Layers for Spin-Polarized Charge Transport and Spin-Dependent Electrocatalytic Applications” *Advanced Science* **2022**, *9*, DOI 10.1002/advs.202201063.

[2] T. Ying, N. Onofrio, L. Mei, H. Peng, Z. Zhang, M. Gu, C. Ma, Y. Chen, J. Zhou, K. M. Y. Leung, C. Cheng, S. Li, B. Liu, C. Y. Tang, D. Voiry, Z. Zeng, “Vacancy Functionalized MoS 2 Nanolaminated Membranes for Efficient Sieving in Forward Osmosis” *Advanced Materials* **2025**, DOI 10.1002/adma.202504781.

[3] W. Wang, N. Onofrio, E. Petit, B. A. Karamoko, H. Wu, J. Liu, J. Li, K. Qi, Y. Zhang, C. Gervais, L. Lajaunie, C. Salameh, P. Miele, Z. Zeng, D. Voiry, “High-surface-area functionalized nanolaminated membranes for energy-efficient nanofiltration and desalination in forward osmosis” *Nature Water* **2023**, *1*, 187–197.
